# Immunogenicity and safety of routine vaccines in children and adolescents with rheumatic diseases on immunosuppressive treatment — a systematic review

**DOI:** 10.1007/s00431-021-04283-w

**Published:** 2021-12-22

**Authors:** Michèle Keller, Laure F. Pittet, Petra Zimmermann

**Affiliations:** 1 Faculty of Science and Medicine, University of Fribourg, Fribourg, Switzerland; 2 Pediatric Infectious Diseases Unit, Division of General Pediatrics, Department of Pediatrics, Gynecology & Obstetrics, Faculty of Medicine, University Hospitals of Geneva and University of Geneva’s, Geneva, Switzerland; 3 Infectious Diseases Group, Murdoch Children’s Research Institute, Parkville, VIC Australia; 4 Department of Paediatrics, The University of Melbourne, Parkville, VIC Australia; 5 Department of Paediatrics, Fribourg Hospital HFR, Fribourg, Switzerland

**Keywords:** Antibodies, Disease activity, DMARD, Humoral response, Immunisation, Juvenile arthritis, Juvenile dermatomyositis, Juvenile autoimmune rheumatic diseases, Systemic lupus erythematosus

## Abstract

The immunogenicity of vaccines in children with juvenile autoimmune rheumatic diseases (JARDs) can be reduced, there are additional safety concerns around vaccination, and there is a potential for worsening in disease activity. In this systematic review, we summarise studies that investigated the immunogenicity and safety of routine vaccines in children and adolescents with JARD on immunosuppressive treatment. We identified 37 studies investigating 2571 children and adolescents with JARD on immunosuppressive treatment and 4895 control children. Of the 56 geometric mean antibody titres measured, 19 (34%) were lower, six (11%) higher, and 31 (55%) similar; of the 39 seroprotection rates measured, 10 (26%) were lower, two (5%) higher, and 27 (69%) similar; and of the 27 seroconversion rates measured, nine (33%) were lower, two (8%) higher, and 16 (59%) similar in children with JARD on immunosuppressive treatment compared with control children. However, many of the studies were underpowered, and not designed to show non-inferiority between children with JARD and controls. Subgroup analysis for different types of immunosuppressive treatments was not feasible, as most studies did not report results by treatment. Severe adverse events were reported in 38 children (33 with juvenile idiopathic arthritis, four with systemic lupus erythematosus, and one in a healthy child); most of them were likely not related to the vaccination (e.g. elective hospitalisation or surgery). A worsening in disease activity was reported in 44 (2%) children with JARD; again, many of them were likely not related to the vaccination. There were no safety concerns with live attenuated vaccines; however, only few studies reported results for this.

*Conclusion*: Vaccination in children with JARD on immunosuppressive treatment is safe and should be promoted, especially since these children are at increased risk for infection. The importance for the completion of vaccination schedules should be stressed. Strategies to compensate for the lower vaccine responses, which are found in approximately one-third of these children, include measuring antibody levels to determine the optimal timing for the administration of additional booster doses.

**Table:** 

**What is Known:**
*• Children with juvenile autoimmune rheumatic diseases (JARDs) are at higher risk for infections, due to their underlying disease and their immunosuppressive treatment.*
*• In children with JARD, the immunogenicity of vaccines might be reduced, and concerns about safety or the potential for worsening in disease activity after vaccination exist.*
**What is New:**
*• Our systematic review shows that vaccines in children with JARDs on immunosuppressive treatment are safe and immunogenic.*
*• There are several limitations of the currently published studies, including random timing of measuring vaccine responses and age differences between children with JARD and control groups. Many of the studies were underpowered, and not designed to show non-inferiority between children with JARD and controls.*

**Supplementary Information:**

The online version contains supplementary material available at 10.1007/s00431-021-04283-w.

## Introduction

Juvenile autoimmune rheumatic diseases (JARDs) are frequent in children and adolescents. The global incidence of juvenile idiopathic arthritis (JIA), for example, is estimated at 1 per 1000 children and has been reported to be increasing over the past decades [1, 2]. Children with JARD are at higher risk for infection due to their underlying disease but also because they are often on immunosuppressive treatment. Vaccination is the most effective and economic method of preventing infectious diseases [3]. Children with JARD are often under-vaccinated and therefore at higher risk for vaccine-preventable diseases, as parents and paediatricians may refuse or delay vaccinations due to safety concerns [4, 5]. Furthermore, compared to healthy children, the immunogenicity of vaccines in children and adolescents with JARD can be reduced and there might be concerns for a potential in worsening in disease activity [6, 7].

The immunogenicity and safety of vaccines in children with JARD have previously been reviewed [8, 9]. However, in the past decade, new immunosuppressive agents have become available for the treatment of JARD in children and additional studies have been published on the immunogenicity of vaccines in this group. Therefore, there is a need of an updated overview on this topic to assure the safe and most beneficial use of vaccines in these children.

In this systematic review, we summarise studies that have investigated the immunogenicity (humoral responses) and safety of vaccines in children and adolescents with JARD on immunosuppressive treatment.

## Systematic review methods

A systematic search was done according to the preferred reporting items for systematic reviews and meta-analyses, the PRISMA guidelines [10]. In March 2021, MEDLINE (1946 to present) and Embase (1947 to present) were searched using the Ovid interface with the following search term combination: “child” AND “vaccination” AND “immunosuppressive treatment” (see supplementary data for detailed search terms). No language limitations were used. We included original studies which investigated the immunogenicity (specific immunoglobulin G responses) and safety of routine vaccines in children and adolescents up to the age of 21 years with JARD on immunosuppressive treatment. Exclusion criteria were studies which (i) did not specify the immunosuppressive treatment, (ii) included children with renal insufficiency or on dialysis, (iii) had less than 10 participants, and (iv) did not report results for children separately to those from adults. References of retrieved articles were hand-searched for additional publications.

The following variables were extracted from the included studies: author, publication year, country, study type, level of evidence, number of participants, age and gender of participants, immunosuppressive treatment, vaccine type, vaccine brand, vaccine producer, vaccine dose, number of vaccine doses, interval between doses, timing of blood sampling after last vaccination, antibody responses, safety (including local and systemic reactions, serious adverse events and worsening in disease activity), and additional important findings. The ROBINS-1 tool was used to assess risk of bias [11].

## Systematic review results

**Fig. 1:**
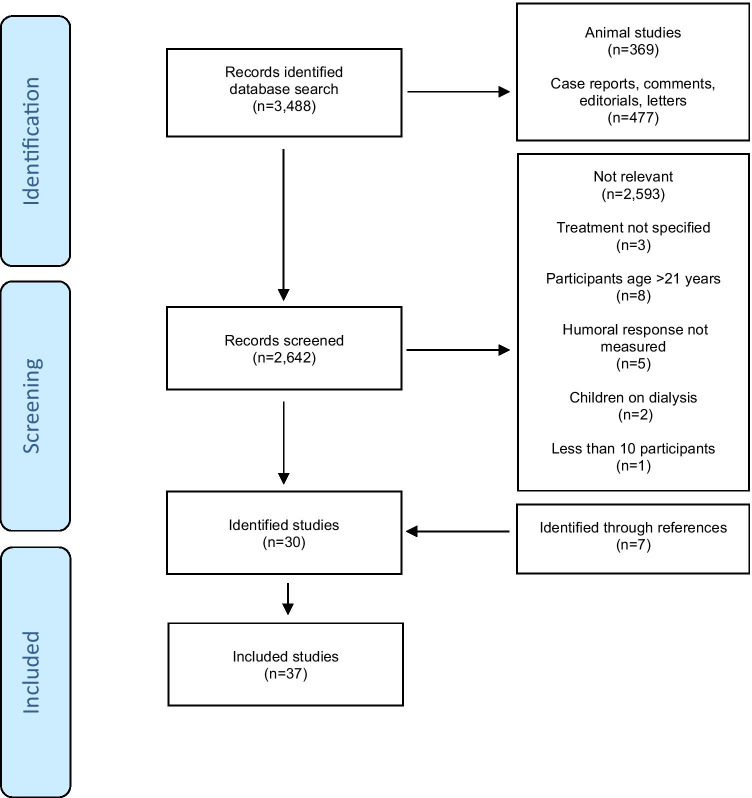
Selection of studies

Our search identified 3488 studies. Of these, 30 fulfilled the inclusion criteria [12–41]. Seven additional studies were found by hand-searching of references [42–48]. The selection of included studies is summarised in Fig. 1. The 37 studies (28 cohort studies, three case–control studies, three cross-sectional studies, two randomised controlled trials (RCTs) and one case series) included in this review investigated 2571 children and adolescents with JARD on immunosuppressive treatment and 4895 control children (4865 healthy children and 30 children with non-rheumatic diseases). The number of participants in each study ranged from 23 to 2576 (median 77, mean 202). Antibodies against 22 different antigens were measured. Of the studies, 30 evaluated the safety of vaccines, 25 local reactions, 26 systemic reactions, 27 severe adverse events (SAEs), and 26 worsening in disease activity. All of the studies were done in industrialised countries: Brazil 11, Netherlands 8, Greece 4, Turkey 3, Germany 2, Japan 2, Iran 1, Italy 1, Slovenia 1, Spain 1, Sweden 1, Switzerland 1, and USA 1. The results of these studies are summarised in Tables 1 and 2 [12–48]. The risk of bias summary of studies included in the review can be found in Table 3.

**Table 1:** Summary of findings of studies investigating the influence of immunosuppressive treatment on humoral vaccine responses in children and adolescents with JARD in comparison to control children (results from last follow-up time-point of each study)

	**HAV**	**HBV**	**HPV**	**Diphtheria**	**Tetanus**	**Influenza A/H1N1**	**Influenza A/H3N2**	**Influenza B**	**MenC**	**Pneumococcus**	**Measles**	**Mumps**	**Rubella**	**Varicella**
**Enthesitis-related arthritis**														
**GMT**											↓^43^		↓^43^	
**SPR**											= ^43^		= ^43^	
**Juvenile dermatomyositis**														
**GMT**						= ^23^								
**SPR**			= ^42^			= ^23^								
**SCR**						↓^23^								
**Juvenile idiopathic arthritis**														
**GMT**	↓^13^	↓^16^	= ^44^	↓^30^	↓^30^	↑^28^ ↓^31^ = ^12,22,29^	↓^28^ = ^12,29,31^	↓^28,31^ = ^12,29^	↓^26^ = ^20^	↓^38^ = ^19^	↑^30,45^ ↓^14^* = ^14^°	↑^45^ ↓^30^ = ^14^	↑^45^ ↓^30^ = ^14^	
**SPR**	↓^35^ = ^13^	↓^16^		↓^30^	↓^30^	= ^12,22,29,31,46^	↑^46^ = ^12,29,31^	↓^31,46^ = ^12,29^		= ^19,38^	↑^30‡^ = ^30†^	↓^30†^ = ^30‡^	↓^30†^ = ^30‡^	
**SCR**	= ^13^					↓^22,28,46^ = ^12,29,31^	↑^46^ ↓^28^ = ^12,29,31^	↑^46^ ↓^28,31^ = ^12,29^		= ^19^				
**Systemic lupus erythematosus**														
**GMT**		↓^34^			↓^36§^ = ^15,36¶^	↓^21^					= ^36^			= ^47^
**SPR**		= ^34^				↓^21^								
**SCR**						↓^21^								
**Juvenile autoimmune rheumatic disease**														
**GMT**			= ^48^		↓^40^	↓^32^ = ^18,33,37^	= ^33,37^	↑^37^ = ^33^			= ^40^		= ^40^	= ^24,25,39^
**SPR**						↓^32^ = ^18,33^	= ^33^	= ^33^						= ^39^
**SCR**						↓^32^ = ^18,33,37^	= ^33,37^	= ^33,37^						

*GMT* geometric mean titre, *HAV* hepatitis A virus, *HBV* hepatitis B virus, *HPV* human papillomavirus, *MenC *meningococcus C*,* *SCR* seroconversion rate, *SPR* seroprotection rate

^¶^Active systemic lupus erythematosus

^§^Inactive systemic lupus erythematosus

°Methotrexate ± etanercept > 4 years after mumps - measles rubella vaccination

^‡^One dose of monovalent measles vaccine followed by measles-mumps-rubella vaccination

^†^Two doses of measles-mumps-rubella vaccination

**Table 2:** Summar﻿y of findings of studies investigating the influence of immunosuppressive treatment on humoral vaccination responses in children and children with JARD

**Author** **Country** **Publication year**	**Number of participants (% female)**, **mean age (unless otherwise specified)** **Treatment** **Number of controls (% female)**, **mean age (unless otherwise specified)** **Treatment (if any)**	**Study type** (**Level of evidence)**	**Vaccine (producer)** **Dosage** **Number of doses (interval)**	**Timing of blood sampling after last vaccination**	**Immunogenicity**	**Local reactions** **Systemic reactions** **Severe adverse events** **Worsening in disease activity**	**Additional findings**
**Enthesitis-related arthritis (ERA)**							
**Measles, rubella**							
Maritsi et al. [43] Greece 2019	41 ERA (25%), 11.7 y (SD 4.3) 41 adalimumab initiated after vaccination, 25 MTX, 6 SSZ^1^ 149 healthy, 11.8 y (SD 3.7)	Single-centre, prospective cohort study (2B)	Measles (NA) NA 2 (age 2 y and 5 y) Rubella (NA) NA 2 (age 2 y and 5 y)	12 m^2^	• Lower GMT for measles in ERA (190.0 (95%CI 138.0–264.0) vs 256.0 IU/mL (95%CI 159.0–294.0), *p* = 0.04) • Lower GMT for rubella in ERA (29.9 (95%CI 23.2–38.5) vs 41.6 IU/mL (95%CI 35.0–46.0), *p* < 0.01) • No difference in SPR for measles and rubella between ERA and controls	NA NA NA NA	No difference in GMT and SPR for measles and rubella for children who were only on adalimumab or on additional MTX or SSZ
				36 m^2^	• Lower GMT for measles in ERA (167.0 (95%CI 124.0–256.0) vs 251.0 IU/mL (95%CI 107.0–264.0), *p* = 0.02) • Lower GMT for rubella in ERA (26.2 (95%CI 20.2–33.9) vs 41.0 IU/mL (95%CI 32.0–46.0), *p* < 0.01) • No difference in SPR for measles and rubella between ERA and controls		No difference in GMT and SPR for measles and rubella for children who were only on adalimumab or on additional MTX or SSZ
**Juvenile dermatomyositis (JDM)**							
**Human papillomavirus**							
Grein et al. [42] Brazil 2020	42 JDM (100%), median 16.0 y (IQR 13.0–19.0) 20 steroids, 17 hydroxychloroquine, 15 MTX, 11 none, 8 azathioprine, 7 cyclosporine, 2 IVIG, 1 mycophenolate mofetil^1^ 35 healthy (100%), median 14.0 y (IQR 6.5–21.5)	Multicentre, prospective cohort study (2B)	Gardasil^®^(NA) NA 3 (0; 1 m or 2 m; 6 m)	1 m	• No difference in SPR for HPV16 and HPV18 between JDM and controls	JDM: pain 57 (47%), induration 14 (12%), oedema 10 (8%), redness 7 (6%), bruise 1 (1%) Controls: pain 65 (59%), oedema 21 (19%), induration 18 (16%), redness 9 (8%), bruise 6 (5%)^3^ JDM: headache 25 (21%), fatigue 14 (12%), nausea 12 (10%), itchiness 11 (9%), muscular pain 4 (3%), new cutaneous abnormalities 4 (3%), articular pain 2 (2%), fever 2 (2%), vomiting 2 (2%) Controls: headache 27 (24%), fatigue 19 (17%), nausea 7 (6%), itchiness 2 (2%), muscular pain 2 (2%), fever 1 (1%)^3^ None JDM: 1 (2%)	No difference in SPR for HPV18 for different treatments 2 JDM (1 on MTX, 1 on hydroxychloroquine + steroids) did not reach seroprotection for HPV16 and HPV18 1 JDM (on azathioprine + hydroxychloroquine + steroids) did not reach seroprotection for HPV18
				6 m	• SPR for HPV16 and HPV18 94% in JDM, values in controls NA		No difference in SPR for HPV16 and HPV18 for different treatments 1 JDM (on azathioprine + hydroxychloroquine + steroids) did not reach seroprotection for HPV 18
**Influenza**							
Guissa et al. [23] Brazil 2012	30 JDM (63%), median 15.5 y (range 9.0–21.0, 7 children ≤ 18 y) 15 steroids, 14 MTX, 6 cyclosporine, 2 azathioprine, 7 chloroquine^1^ 81 healthy (41%), median 15.0 y (range 9.0–21.0)	Single-centre, prospective cohort study (2B)	NYMCx-179A (*Butantan Institute/Sanofi Pasteur*) • A/California/7/2009(H1N1)pdm09 15 μg 1	21 d	• Lower SCR in JDM (87 (95%CI 75–99) vs 98% (95%CI 94–100), *p* = 0.04) • No difference in GMT and SPR between JDM and controls	JDM: pain 9 (30%) Controls: pain 19 (23%), swelling 2 (2%), redness 2 (2%), itching 1 (1%) JDM: headache 5 (17%), rhinorrhoea 4 (13%), arthralgia 1 (3%), fever 1 (3%), myalgia 1 (3%) Controls: headache 18 (22%), myalgia 6 (7%), cough 5 (6%), sore throat 5 (6%), fever 3 (4%), rhinorrhoea 3 (4%), nasal congestion 3 (4%), arthralgia 2 (2%), diarrhoea 2 (2%) None None	4 JDM did not reach seroprotection
**Juvenile idiopathic arthritis (JIA)**							
**Hepatitis A**							
Maritsi et al. [13] Greece 2017	83 JIA (66%), 6.3 y (SD 2.3) 83 MTX 76 healthy (45%), 5.3 y (SD 2.7)	Single-centre, prospective cohort study (2B)	Havrix^®^(*GlaxoSmithKline*) 720 IU/mL 2 (0; > 6 m)	1 m	• Lower GMT in JIA (94.0 vs 162.5 mIU/mL, *p* < 0.01) • No difference in SCR and SPR between JIA and controls	JIA: 5 (6%) Controls: 4 (5%) JIA: fever 3 (4%), malaise 3 (4%) Controls: fever 2 (3%), malaise 2 (3%) None JIA: 15 (18%) (in 2 after the 1st dose and in 13 after the 2nd dose after a mean of 8 months)	No difference in GMT, SCR and SPR between different JIA subtypes (ERA, oligoarticular JIA with, polyarticular JIA, psoriatic JIA) No difference in GMT, SCR and SPR in JIA with or without presence of uveitis or ANA positivity
				12 m	• Lower GMT in JIA (98.2 vs 200.3 mIU/mL, *p* < 0.01) • No difference in SCR and SPR between JIA and controls		
Erguven et al. [35] Turkey 2011	47 JIA (51%), 10.7 y (SD 3.9) 19 SSZ, 18 MTX, 11 MTX + steroids, 4 etanercept, 1 steroids^1^ 67 healthy (46%), 9.4 y (SD 3.8)	Single-centre, prospective cohort study (2B)	Hepatitis A (NA) NA 2 (0; 6 m)	2 m	• Lower SPR in JIA (92 vs 100%, *p* < 0.05)	None None None None	
**Hepatitis B**							
Kasapçopur et al. [16] Turkey 2004	39 JIA (46%), 10.0 y (SD 3.3, range 4.0–16.0) 19 MTX, 17 steroids, 3 MTX + steroids 41 healthy (51%), 8.8 y (SD 2.6, range 5.0–14.0)	Single-centre, prospective cohort study (2B)	Engerix-B^®^(*GlaxoSmithKline*) 10 μg (weight < 20 kg), 20 μg (weight > 20 kg) 3 (0; 1 m; 3 m or 6 m)	1 m	• Lower GMT in JIA (137.4 vs 258.9 mIU/mL, *p* = NA) • Lower SPR in JIA (97 vs 100%, *p* = NA)	None None None NA	No difference in GMT for different treatments No difference in GMT for different vaccine schedules (3rd dose at 3 or 6 m)
**Human papillomavirus**							
Heijstek et al. [44] Netherlands 2014	68 JIA (100%), 14.1 y (SD 1.6) 24 MTX, 9 anti-TNF-alpha, 5 leflunomide, 1 anti-IL-1, 1 mycophenolate mofetil^1^ 55 healthy (100%), 14.3 y (SD 1.2)	Single-centre, prospective cohort study (2B)	Cervarix^®^(*GlaxoSmithKline*) NA 3 (0; 1 m; 6 m)	1 m	• No difference in GMT between JIA and controls	JIA: pain 52 (96%), induration 26 (48%), swelling 25 (46%) redness 20 (37%), bruising 14 (26%) Controls: pain 44 (100%), redness 43 (98%), bruising 39 (89%), induration 21 (48%), swelling 18 (41%) JIA: fatigue 30 (56%), myalgia 29 (54%), headache 22 (41%), arthralgia 11 (20%), rash 11 (20%), fever 6 (11%), syncope 1 (2%) Controls: fatigue 22 (50%), headache 22 (50%), myalgia 19 (43%), arthralgia 6 (14%), rash 6 (14%), fever 3 (7%) JIA: endoscopies 3 (4%), pre-planned surgeries 2 (3%), allergic reaction to anti-TNF-alpha 1 (1%), analyse of gait abnormalities 1 (1%), appendicitis 1 (1%), correction of cerebral arteriovenous malformation 1 (1%), epileptic insult (cerebral arteriovenous malformation) 1 (1%), perforated eardrum 1 (1%), pre-planned laser therapy uveitis 1 (1%), severe pharyngitis 1 (1%) transient lower back pain 1 (1%), Controls: pre-planned surgery 1 (2%) None	1 JIA (on MTX) did not reach seroprotection at 6 m No difference in GMT between JIA on MTX and JIA on other treatments
				6 m	• No difference in GMT between JIA and controls • No difference in avidity of HPV16/18-specific IgGs between JIA and controls		
**Diphtheria, tetanus**							
Brunner et al. [41] USA 2020	29 JIA (55%), 4.2 y (SD 0.9) 29 abatacept, 22 MTX, 3 steroids^1^	Multicentre, cross-sectional study (2C)	Diphtheria and tetanus (NA) NA NA	NA	• SPR for diphtheria in JIA 90% • SPR for tetanus in JIA 100%	NA NA NA NA	3 JIA did not reach seroprotection No difference in SPR for different treatments No difference in SPR for different vaccine types or schedule
Heijstek et al. [30] Netherlands 2012	400 JIA (NA), 9.4 y (SD 4.5) 93 MTX, 28 steroids, 8 anti-TNF-alpha 2176 healthy (NA), 7.9 y (SD 5.5)	Single-centre, cross-sectional (2C)	Diphtheria and tetanus (*Dutch National Institute of Public Health and the Environment/the Dutch Vaccine Institute*) NA 6 (4 doses until age 1 y; 4 y; 9 y)	NA	• Lower GMT for diphtheria in JIA (NA, *p* < 0.01) • Lower GMT for tetanus in JIA (NA, *p* < 0.01) • Lower SPR for diphtheria in JIA (91 vs 99%, *p* < 0.01) • Lower SPR for tetanus in JIA (96 vs 99%, *p* < 0.01)	NA NA NA NA	No difference in GMT between JIA on MTX and JIA on other treatments No difference in GMT between JIA on steroids and JIA without steroids
				NA	• Lower GMT for diphtheria in JIA (NA, *p* < 0.01) • Lower GMT for tetanus in JIA (NA, *p* < 0.01) • Lower SPR for diphtheria in JIA (77 vs 99%, *p* < 0.01) • Lower SPR for tetanus in JIA (94 vs 99%, *p* = 0.01)		
**Influenza**							
Camacho-Lovillo et al. [12] Spain 2017	35 JIA (66%), median 10.6 y (IQR 8.8–12.7) 11 etanercept, 4 anakinra, 4 tocilizumab, 4 adalimumab (7 additional MTX), 7 MTX, 2 tocilizumab + MTX + steroids, 3 none 6 healthy (67%), median 11.6 y (IQR 9.8–14.6)	Single-centre, prospective cohort study (2B)	TIV (*Sanofi Pasteur MSD*) • A/California/7/2009 (H1N1)pdm09 • A/Victoria/361/2011(H3N2) • B/Massachusetts/2/2012 NA 1 (> 9 y), 2 (< 9 y) (0; 1 m)	1–2 m	• No difference in GMT, SCR, SPR for any of the 3 strains between JIA and controls	Whole cohort^4^: local skin inflammation 6 (15%), haematoma 1 (2%) JIA: general malaise and fever > 24 h 1 (3%) Controls: general malaise and fever > 24 h 1 (17%) None JIA: 6 (17%)	No difference in GMT, SCR, SPR for any of the 3 strains for different biologicals or additional steroids
				12 m	• No difference in GMT, SCR, SPR for any of the 3 strains between JIA and controls		No difference in GMT, SCR, SPR for any of the 3 strains for different biologicals or additional steroids
Aikawa et al. [22] Brazil 2013	95 JIA (56%), 14.9 y (SD 3.2) 55 steroids, leflunomide, cyclosporine or SSZ, 47 MTX, 16 anti-TNF-alpha^1^ 91 healthy (52%), 14.6 y (SD 3.7)	Single-centre, prospective cohort study (2B)	NYMCx-179A (*Butantan Institute/Sanofi Pasteur*) • A/California/7/2009 (H1N1)pdm09 15 μg 1	21 d	• Lower SCR in JIA (83 (95%CI 76–91) vs 96% (95%CI 91–100), *p* < 0.01) • No difference in GMT and SPR between JIA and controls	JIA: local pain 20 (21%) Controls: local pain 21 (23%) JIA: myalgia 15 (16%), headache 14 (15%) Controls: headache 18 (20%), myalgia 6 (7%) None None	Lower SCR in JIA with polyarticular disease^5^ compared to controls (80 (95%CI 68–92) vs 96% (95%CI 91–100, *p* < 0.01) Lower SCR in JIA without anti-TNF-alpha compared to controls (81 (95%CI 72–90) vs 96% (95%CI 91–100), *p* < 0.05) Lower SPR in JIA without anti-TNF-alpha compared to controls (86 (95%CI 78–94) vs 96% (95%CI 91–100), *p* < 0.05) No difference in GMT for different treatments
Carvalho et al. [46] Brazil 2013	44 JIA (NA), 11.0 y 31 leflunomide or MTX, 6 steroids, 5 anti-TNF-alpha, 1 cyclosporine 10 healthy (NA), mean NA (range 3.0–18.0)	Single-centre, prospective cohort study (2B)	TIV (*Butantan Institute/Sanofi Pasteur SA)* • A/Solomon/3/2006(H1N1) • A/Brisbane/10/2007(H3N2) • B/Florida/4/2006 0.25 mL (1–3 y), 0.5 mL (> 3 y) 1 (> 9 y), 2 (< 9 y) (0; 1 m)	30–40 d	• Lower SCR for A/H1N1 strain in JIA (93 vs 100%, *p* = NA) • Higher SCR for A/H3N2 strain in JIA (74 vs 38%, *p* = NA) • Higher SCR for B strain in JIA (78 vs 75%, *p* = NA) • Higher SPR for A/H3N2 strain in JIA (91 vs 80%, *p* = NA) • Lower SPR for B strain in JIA (95 vs 100%, *p* = NA) • No difference in SPR for A/H1N1 strain between JIA and controls	JIA: pain 6 (14%), redness and swelling 2 (7%) Controls: pain 1 (10%), redness and swelling 1 (10%) JIA: rhinorrhoea and cough 6 (14%) Controls: None None None	Lower SCR for A/H1N1 strain in JIA on anti-TNF-alpha compared to other treatments (*p* = 0.03) Lower SPR for A/H1N1 strain in JIA on anti-TNF-alpha compared to other treatments (*p* = NA) No difference in SPR for A/H3N2 and B strains between JIA on anti-TNF-alpha and controls
Dell’Era et al. [31] Italy 2012	30 JIA (73%), 8.4 y (SD 4.6) 30 MTX or SSZ 30 JIA (47%), 9.5 y (SD 5.7) 30 etanercept 30 healthy (50%), 9.1 y (SD 5.0)	Single-centre, prospective cohort study (2B)	TIV (*Novartis*) • A/California/7/2009(H1N1)pdm09 • A/Perth/16/2009(H3N2) • B/Brisbane/60/2008 15 μg 1	1 m	• Lower GMT for A/H1N1 strain in JIA on etanercept compared to JIA on MTX and controls (773.6 vs 1833.8 vs 1693.1, *p* < 0.05) • Lower GMT for B strain in JIA on etanercept compared to JIA on MTX and controls (61.2 vs 187.7 vs 210.6, *p* < 0.05) • Lower SCR for B strain in JIA on etanercept compared to JIA on MTX and controls (30 vs 83 vs 93%, *p* < 0.05) • Lower SPR for B strain in JIA on etanercept compared to JIA on MTX and controls (30 vs 83 vs 93%, *p* < 0.05) • No difference in GMT for A/H3N2 strain between JIA on etanercept, JIA on MTX or SSZ and controls • No difference in SCR and SPR for A/H1N1 and A/H3N2 strains between JIA on etanercept, JIA on MTX or SSZ and controls	JIA on MTX or SSZ: pain 13 (43%), swelling 12 (40%), erythema 2 (7%) JIA on etanercept: pain 11 (37%), swelling 11 (37%), erythema 3 (10%) Controls: pain 12 (40%), swelling 11 (37%), erythema 3 (10%) JIA on MTX or SSZ: rhinitis 9 (30%), changing eating habits 8 (27%), fever 7 (23%), malaise 6 (20%), sleepiness 6 (20%), diarrhoea 2 (7%), vomiting 2 (7%) JIA on etanercept: malaise 8 (27%), rhinitis 8 (27%), changing eating habits 4 (13%), fever 4 (13%), sleepiness 4 (13%), vomiting 2 (7%), diarrhoea 1 (3%) Controls: malaise 8 (27%), rhinitis 7 (23%), changing eating habits 5 (17%), fever 5 (17%), sleepiness 5 (17%), diarrhoea 2 (7%), vomiting 1 (3%) JIA on etanercept: hospitalisation for fever and coxalgia 1 (3%) Controls: none JIA on MTX: 1 (3%)	
				3 m	• Lower GMT for A/H1N1 in JIA on etanercept compared to JIA on MTX and controls (463.4 vs 1067.7 vs 1324.5, *p* < 0.05) • Lower GMT for B strain in JIA on etanercept compared to JIA on MTX and controls (33.3 vs 162.6 vs 193.3, *p* < 0.05) • Lower SCR for B strain in JIA on etanercept compared to JIA on MTX and controls (10 vs 83 vs 90%, *p* < 0.05) • Lower SPR for B strain in JIA on etanercept compared to JIA on MTX and controls (10 vs 83 vs 90%, *p* < 0.05) • No difference in GMT for A/H3N2 strain between JIA on etanercept, JIA on MTX or SSZ and controls • No difference in SCR and SPR for A/H1N1 and A/H3N2 strains between JIA on etanercept, JIA on MTX or SSZ and controls		
Shinoki et al. [29] Japan 2012	27 JIA (48%), 10.4 y (SD 5.6) 24 steroids + tocilizumab, 3 tocilizumab 17 healthy (47%), 10.6 y (SD 6.2)	Single-centre, prospective cohort study (2B)	TIV (NA) • A/Solomon/3/2006(H1N1) • A/Hiroshima/52/2005(H3N2) • B/Malaysia/2506/2004 0.2 (1–6 y), 0.3 (6–13 y), 0.5 mL (> 13 y) 1 (> 13 y), 2 (< 13 y) (0; 7–28 d)	28–49 d	• No difference in GMT, SCR and SPR for any of the 3 strains between JIA and controls	JIA: local erythema and swelling 3 (11%), local induration 1 (4%) Controls: none JIA: influenza with fever and rhinorrhoea 1 (4%) Controls: none None NA	No difference in GMT for any of the 3 strains between JIA on tocilizumab since ≥ 2 y and JIA on < 0.2 mg/kg steroids No difference in GMT for A/H3N2 and B strains between JIA on > 0.2 mg/kg steroids and JIA on < 0.2 mg/kg steroids
Toplak et al. [28] Slovenia 2012	31 JIA (68%), 11.0 y (SD 4.5, range 3.0–18.0) 18 none, 8 MTX, 3 leflunomide, 2 SSZ, 7 steroids plus MTX, leflunomide or SSZ, 3 etanercept plus MTX, leflunomide or SSZ, 1 infliximab plus MTX, leflunomide or SSZ 14 children with cardiac diseases (29%), 11.9 y (SD 4.5, range 4.0–18.0)	Single-centre, prospective cohort study (2B)	Begrivac 2008/2009^®^(*Novartis*) • A/Brisbane/59/2007(H1N1) • A/Brisbane/10/2007(H3N2) • B/Florida/4/2006 15 μg 1, 2 (< 9 y + 1st dose) (0; 1 m)	1 m	• Lower GMT for A/H1N1 strain in JIA (174.9 vs 240.7, *p* = NA) • Lower GMT for A/H3N2 strain in JIA (153.2 vs 158.7, *p* = NA) • Higher GMT for B strain in JIA (100.2 vs 92.2, *p* = NA) • Lower SCR for A/H1N1, A/H3N2, B strains in JIA (68 vs 79%, *p* = NA)	JIA: pain 10 (32%) Controls: pain 3 (21%) JIA: malaise and headache 1 (3%) Controls: malaise 2 (14%) None JIA: 11 (35%) 1 m after vaccination 4 2 m after vaccination 1 6 m after vaccination 6	2 JIA and 1 control did not reach seroprotection for A/H1N1 strain 6 JIA and 3 controls did not reach seroprotection for A/H3N2 strain 5 JIA and 3 controls did not reach seroprotection for B strain
				6 m	• Higher GMT for A/H1N1 strain in JIA (90.9 vs 72.7, *p* = NA) • Lower GMT for A/H3N2 strain in JIA (86.3 vs 88.5, *p* = NA) • Lower GMT for B strain in JIA (98.3 vs 113.5, *p* = NA) • Lower SCR for A/H1N1, A/H3N2, B strains in JIA (77 vs 79%, *p* = NA)		Higher GMT for A/H1N1 (105.9 vs 90.9 vs 72.7, *p* = NA), A/H3N2 (95.4 vs 86.3 vs 88.5, *p* = NA) and B strains (113.8 vs 98.3 vs 113.5, *p* = NA) in JIA on MTX, leflunomide or SSZ compared to JIA on other treatments and controls
**Meningococcus C**							
Stoof et al. [20] Netherlands 2014	127 JIA (62%), 8.9 y (SD 3.7) 108 MTX, 44 etanercept, 14 steroids, 6 infliximab, 9 anakinra, 1 anti-IL-6 1527 healthy (54%), 9.1 y (SD 5.1)	Single-centre, retrospective cohort study (2B)	NeisVac-C^®^(*Baxter Healthcare*) 19 μg (*N. meningitidis* serogroup C polysaccharide strain C11), 10–20 μg (tetanus toxoid) 1	50 m	• No difference in GMT between JIA and controls	NA NA NA NA	No difference in GMT decrease in JIA starting MTX treatment during the study
Zonneveld-Huijssoon et al. [26] Netherlands 2007	234 JIA (65%), 11.1 y (SD 4.2, range 1.5–18.9) Group I: 47 none Group II: 41 NSAID Group III: 36 MTX, 7 SSZ Group IV: 15 MTX, 6 etanercept, 2 infliximab, 2 MTX + SSZ, 1 cyclosporine	Multicentre, prospective cohort study (2B)	NeisVac-C^®^(NA) 20 μg/mL (*N. meningitides* Z2491 serogroup C polsysaccharide), 20–40 μg/mL (tetanus toxoid) 1	< 3 m	• Lower GMT in group III and IV compared to group I and II (17.5 vs 16.3 vs 41.0 vs 46.9 μg/mL, *p* ≤ 0.01) • No difference in GMT between group III and group IV	NA NA NA None	Low GMT (< 2 µg/mL) in 4 JIA (2 on etanercept + MTX, 1 on MTX, 1 on SSZ)
**Pneumococcus**							
Aikawa et al. [19] Brazil 2015	17 JIA (47%), median 11.6 y 17 etanercept, 16 MTX, 6 steroids, 4 cyclosporine, 4 leflunomide^1^ 10 JIA (40%), median 9.2 y 10 MTX, 1 cyclosporine^1^	Single-centre, prospective cohort study (2B)	Pneumovax^®^(*Sanofi Pasteur*) NA 1	2 m	• No difference in GMT, SCR, SPR for serotypes 4, 6B, 9 V, 14, 18C, 19F, 23F between JIA on etanercept and controls	JIA on etanercept: none Controls: swelling and redness 1 (10%) JIA on etanercept: upper respiratory tract infection requiring antibiotics 11 (65%) Controls: upper respiratory tract infection requiring antibiotics 3 (30%) JIA on etanercept: invasive pneumococcal disease with a bacterial pneumonia requiring hospitalization 1 (6%) Controls: none None	
				12 m	• No difference in GMT, SCR, SPR for serotypes 4, 6B, 9 V, 14, 18C, 19F, 23F between JIA on etanercept and controls		
Farmaki et al. [38] Greece 2010	31 JIA (68%), 12.9 y (SD 4.6) 26 MTX, 21 etanercept, 10 adalimumab, 8 steroids, 5 cyclosporine^1^ 32 JIA (78%), 11.6 y (SD 3.3) 26 MTX, 8 steroids, 7 cyclosporine^1^	Single-centre, prospective cohort study (2B)	Prevenar^®^(NA) NA 2 (0; 42–56 d)	Mean 42 d (SD 3.3) after 1st dose	• Lower GMT for serotypes 4 (1.8 vs 5.0 μg/mL, *p* < 0.01), 14 (5.5 vs 10.3 μg/mL, *p* = 0.01) and 23F (4.5 vs 11.2 μg/mL, *p* = 0.02) in JIA on etanercept or adalimumab • No difference in SPR for serotypes 4, 6 V, 9 V, 14, 18C, 19F, 23F	JIA on etanercept or adalimumab 6 (19%): pain, redness, swelling Controls 5 (16%): pain, redness, swelling None None JIA on etanercept or adalimumab: 1 (3%)	No difference in GMT and SPR for JIA on adalimumab compared to JIA on etanercept
**Mumps, measles, rubella**							
Heijstek et al. [45] Netherlands 2013	Group 1: 63 JIA (73%), 6.3 y (95%CI 5.9–6.7) 29 MTX, 5 etanercept, 3 anti-IL-1, 2 steroids, 1 adalimumab, 1 leflunomide^1^ Group 2: 68 JIA (60%), 6.5 y (95%CI 6.2–6.9) 31 MTX, 4 etanercept, 1 anti-IL-1, 1 leflunomide, 1 steroids^1^	Multicentre, non-blinded, controlled, randomised trial (1B)	MMR-NV1^®^(*Netherlands vaccine Institute)* or M-M-RVAXPRO^®^(*Sanofi Pasteur*) NA 1 MMR booster in group 1	12 m	• Higher GMT for measles in JIA with MMR booster (1.6 vs 0.8 IU/mL, *p* = 0.03) • Higher GMT for mumps in JIA with MMR booster (168.0 vs 104.0 RU/mL, *p* = 0.03) • Higher GMT for rubella in JIA with MMR booster (69.0 vs 45.0 IU/mL, *p* = 0.01) • Higher SPR for measles in JIA with MMR booster (100 vs 92% (95%CI 84–99), *p* = ns) • Higher SPR for mumps in JIA with MMR booster (97 (95%CI 95–100) vs 81% (95%CI 72–93), *p* = ns) • Higher SPR for rubella in JIA with MMR booster (100 vs 94% (95%CI 86–100), *p* = ns)	NA Group 1: abdominal pain and/or obstipation 4 (6%), bone fracture due to trauma 4 (6%), upper respiratory tract infection 4 (6%), arthralgia 3 (5%), contusion due to trauma 3 (5%), gastroenteritis 3 (5%), otitis 3 (5%), rash 3 (5%), eczema 2 (3%), fever 2 (3%), molluscum contagiosum 2 (3%), bronchopneumonia 1 (2%), CRP elevation 1 (2%), fungal infection 1 (2%), headache 1 (2%), varicella infection 1 (2%), worsening of pre-existent anxiety attacks 1 (2%) Group 2: contusion due to trauma 4 (6%), gastroenteritis 4 (6%), abdominal pain and/or obstipation 3 (4%), rash 3 (4%), upper respiratory tract infection 3 (4%), viral infection 3 (4%), eczema 2 (3%), myalgia 2 (3%), A/H1N1 infection 1 (1%), fever 1 (1%), headache 1 (1%), impetigo 1 (1%), molluscum contagiosum 1 (1%), vaginal discharge 1 (1%), watery discharge from the eyes 1 (1%) Group 1: hospital admissions 3 (5%), surgery 2 (3%) Group 2: surgery 6 (9%), hospital admission 5 (7%) None	No difference in GMT at 3 and 12 m in JIA on MTX and JIA on other treatments 5 controls were not seroprotected for measles 2 JIA (1 on MTX, 1 on MTX + etanercept) and 12 controls did not reach seroprotection for mumps 4 controls did not reach seroprotection for rubella
Heijstek et al. [30] Netherlands 2012	400 JIA (NA), 9.4 y (SD 4.5) 93 MTX, 28 steroids, 8 anti-TNF-alpha (NA)^1^ 2176 healthy (NA), 7.9 y (SD 5.5)	Single-centre, cross-sectional study (2C)	MMR (*Dutch National Institute of Public Health and the Environment/the Dutch Vaccine Institute)* NA 2 (age 14 m and 9 y)	NA	• Higher GMT for measles in JIA (NA, *p* < 0.01) • Lower GMT for mumps in JIA (NA, *p* < 0.01) • Lower GMT for rubella in JIA (NA, *p* < 0.01) • Higher SPR for measles in JIA (94 vs 87%, *p* = 0.01) • No difference in SPR for mumps between JIA and controls • No difference in SPR for rubella between JIA and controls	NA NA NA NA	Higher GMT for measles (*p* = 0.02) in JIA with high disease activity No difference in GMT between JIA on MTX and JIA on other treatments No difference in GMT between JIA on steroids and JIA without steroids
			Monovalent measles (NA) vaccine plus MMR (NA) or rubella (*Dutch National Institute of Public Health and the Environment*) NA 2 (age 14 m and 4 y or 11 y)	NA	• Higher GMT for measles in JIA (NA, *p* < 0.01) • Lower GMT for mumps in JIA (NA, *p* < 0.01) • Lower GMT for rubella in JIA (NA, *p* < 0.01) • Lower SPR for mumps in JIA (81 vs 95%, *p* < 0.01) • Lower SPR for rubella in JIA (81 vs 99%, *p* < 0.01) • No difference in SPR for measles between JIA and controls		
Borte et al. [14] Germany 2009	15 JIA (NA) Group I: 5 MTX start > 4 y after MMR, 15.8 y (range 14.2–17.9) Group II: 5 MTX ≥ 6 m before MMR, 6.7 y (range 6.6–6.7) Group III: 5 etanercept + MTX ≥ 6 m before MMR, 6.3 y (range 6.0–6.7) Group IV: 22 healthy (NA), 11.2 y (range 1.0–20.0)	Single-centre, prospective nested case–control study (3B)	MMR (NA) NA 2 (age 13–24 m and 6 y)	Group I: 6 m after MTX start	• Lower GMT for measles in JIA (group I) compared to healthy children (group V) (194.3 (IQR 0.0–410.0) vs. 1231.7 IU/mL (IQR 461.0–1730.0), *p* < 0.05) • No difference in GMT for mumps between JIA (group I) and controls • No difference in GMT for rubella between JIA (group I) and controls	NA NA NA None	
				Group II, III, IV: 6 m after vaccination	• No difference in GMT for measles, mumps and rubella between JIA (group II, III) and controls (group IV)		
**Systemic lupus erythematosus (SLE)**							
**Hepatitis B**							
Aytac et al. [34] Turkey 2011	20 SLE (80%), 13.2 y (SD 2.6, range 9.1–19.8) 17 steroids, 11 azathioprine, 3 mycophenolate mofetil, 3 none, 2 hydroxychloroquine^1^ 24 healthy (50%), 8.8 y (SD 2.7, range 5.0–14.0)	Single-centre, prospective cohort study (2B)	Engerix-B^®^(*GlaxoSmithKline*) 10 μg (weight < 20 kg), 20 μg (weight > 20 kg) 3 (0; 1; 6 m)	1 m	• Lower GMT in SLE (310.4 (range 0.0–500.0) vs 618.5 IU/mL (range 70.0–900.0), *p* = NA) • No difference in SPR between SLE and controls	None None None SLE: 3 (15%)	
**Tetanus**							
Miyamoto et al. [36] Brazil 2011	20 inactive SLE (85%), 14.0 (SD 2.0; range 10.0–17.0) 17 chloroquine, 14 steroids, 8 azathioprine, 2 cyclosporine, 2 MTX, 1 mycophenolate mofetil^1^ 10 active SLE (90%), 12.0 (SD 2.0; range 8.0–14.0) 14 steroids, 8 chloroquine, 6 azathioprine, 2 cyclophosphamide^1^ 14 healthy (71%), 14.0 y (SD 3.0, range: 8.0–18.0)	Single-centre, retrospective case–control (3B)	DTP (NA) NA 5 (age 2, 4, 6, 15 m and 4–6 y)	NA	• Lower GMT in inactive SLE (0.14 vs 0.68 IU/mL, *p* < 0.05) • No difference in GMT between active SLE and controls	NA NA NA NA	
Kashef et al. [15] Iran 2008	40 SLE (80%), 14.1 y (range 7.0–21.0) 13 azathioprine + steroids, 10 cyclophosphamide + steroids, 8 mycophenolate mofetil + steroids, 5 azathioprine + cyclophosphamide + steroids 60 healthy (69%), 14.4 y (range NA)	Multicentre, retrospective case–control study (3B)	Tetanus (NA) NA 5 (until age 6 y)	NA	• No difference in GMT between SLE and controls	NA NA SLE: disseminated infections requiring hospital admission 4 (10%) Controls: none NA	
**Influenza**							
Campos et al. [21] Brazil 2013	118 SLE (77%), 16.0 y (SD 3.5) 92 antimalarials, 83 steroids, 44 azathioprine, 15 mycophenolate mofetil, 14 MTX, 3 cyclophosphamide, 2 cyclosporine^1^ 102 healthy (50%), 15.9 y (SD 4.5)	Single-centre, prospective cohort study (2B)	NYMCX-179A (*Butantan Institute/Sanofi Pasteur*) • A/California/7/2009(H1N1) pdm09 15 μg 1	21 d	• Lower GMT in SLE (90.8 (95%CI 67.8–121.7) vs 237.3 NA (95%CI 188.8–298.3), *p* < 0.01) • Lower SCR in SLE (64 (95%CI 54–72) vs 91% (95%CI 84–96), *p* < 0.01) • Lower SPR in SLE (74 (95%CI 65–81) vs 95% (95%CI 89–98), *p* < 0.01)	SLE: itching 20 (17%), redness 13 (11%) Controls: redness 2 (2%) SLE: arthralgia 20 (17%), rhinorrhoea 15 (13%) Controls: rhinorrhoea 4 (4%), arthralgia 1 (1%) None None	Children with SLE on higher steroid doses (18.0 mg/d (SD 21.4)) did more frequently not seroconvert than children with SLE on lower doses (10.5 mg/d (SD 12.5)) Lower frequency of renal involvement in SLE 4 m after-vaccination compared to baseline (28 vs 51%, *p* < 0.01) Higher frequency of mucocutaneous lesions in SLE 4 m after-vaccination compared to baseline (17 vs 6%, *p* = 0.02)
**Measles**							
Miyamoto et al. [36] Brazil 2011	20 inactive SLE (85%), 14.0 (SD 2.0; range 10.0–17.0) 17 chloroquine, 14 steroids, 8 azathioprine, 2 cyclosporine, 2 MTX, 1 mycophenolate mofetil^1^ 10 active SLE (90%), 12.0 (SD 2.0; range 8.0–14.0) 14 steroids, 8 chloroquine, 6 azathioprine, 2 cyclophosphamide^1^ 14 healthy (71%), 14.0 y (SD 3.0, range: 8.0–18.0)	Single-centre, retrospective case–control (3B)	Measles (NA) NA 2 (after age 2 y)	NA	• No difference in GMT between active SLE, inactive SLE and controls	NA NA NA NA	Lower IgM titres in inactive SLE (1.2 vs 1.7 g/l, *p* = 0.03) No difference in IgA and IgG titres between active and inactive SLE and controls
**Varicella**							
Barbosa et al. [47] Brazil 2012	28 SLE (75%), 15.3 y (SD 2.5, range 9.9–18.8) 27 chloroquine, 18 steroids, 9 azathioprine, 2 MTX^1^ 28 healthy (75%), 15.0 y (SD 2.5, range 10.1–18.7)	Multicentre, blinded, controlled, randomised trial (1B)	Oka strain (*Biken*) > 10^3^ PFU 1	1 m	• No difference in GMT between SLE and controls	SLE 2 (7%): NA Controls 6 (21%): NA SLE: headache 10 (36%), fever 3 (11%), rash 1 (4%) Controls: headache 6 (21%), fever 4 (14%), vomiting 1 (4%), rash 1 (4%) None None	
				6 m	• No difference in GMT between SLE and controls		
				12 m	• No difference in GMT between SLE and controls		
**Juvenile autoimmune rheumatic disease (JARD)**							
**Hepatitis A and B**							
Belderok et al. [17] Netherlands 2013	78 JARD (71 JIA, 2 SLE, 2 uveitis, 1 autoinflammatory syndrome, 1 JDM, 1 panuveitis) (64%), median 12.0 y (IQR 9.0–14.0) 42 MTX, 24 MTX + anti-TNF-alpha (adalimumab, etanercept or infliximab), 2 MTX + anti-TNF-alpha + steroids, 4 anti-TNF-alpha, 1 anakinra, 1 anti-TNF-alpha + cyclosporine, 1 azathioprine, 1 cyclosporine, 1 MTX + anakinra, 1 MTX + steroids, 1 mycophenolate mofetil, 1 mycophenolate mofetil + steroids	Multicentre, prospective cohort study (2B)	Ambirix^®^(*GlaxoSmithKline*) 720 ELISA units (HAV), 20 μg (hepatitis B surface antigen) 2 (0; 6–7 m)	35 d	• GMT for HAV in JARD (288.0 mIU/mL) • GMT for HBV in JARD (321.0 mIU/mL) • Positive SCR for HAV in JARD (100% (95%CI 96–100)) • Positive SCR for HBV in JARD (93% (95%CI 86–98))	NA NA NA NA	No difference in GMT for different treatments
**Human papillomavirus**							
Heijstek et al. [48] Netherlands 2013	12 JARD (6 JDM, 6 SLE) (100%) age JDM vs SLE 15.3 (SD 2.3) vs 15.0 (SD 1.5) 6 steroids, 5 none, 2 hydroxychloroquine, 2 MTX, 1 azathioprine, 1 mycophenolate mofetil^1^ 49 healthy (100%), 14.3 y (SD 1.2)	Single-centre, prospective cohort study (2B)	Cervarix^®^(*GlaxoSmithKline*) NA 3 (0; 1; 6 m)	1 m	• Lower GMT for HPV16 (NA, *p* < 0.01) and HPV18 (NA, *p* = 0.04) in JARD	NA NA NA JDM 1 (17%)	1 JDM did not seroconvert (without immunosuppressive drugs)
				6 m	• No difference in GMT between JARD and controls		
**Tetanus**							
Ingelman-Sundberg et al. [40] Sweden 2016	50 JARD (46 JIA, 1 erythema nodosum with arthritis, 1 JDM, 1 mixed connective tissue disease, 1 polyarteritis nodosa) (64%) median age: JARD on MTX 12.2 y (range 6.0–16.0); anti-TNF-alpha + MTX 13.1 y (range 2.9–18.3); no treatment 13.8 y (range 4.3–16.0) 15 etanercept, 10 MTX, 8 adalimumab + MTX, 8 none, 7 infliximab + MTX, 2 golimumab + MTX (some on additional steroids) 31 healthy (32%), median 11.5 y (range 2.4–17.7)	Single-centre, cross-sectional study (2C)	DTP (NA) NA ≥ 3 doses^6^	NA	• Lower GMT for tetanus following booster in JARD on anti-TNF-alpha + MTX or MTX compared to JARD without treatment and healthy controls (NA, *p* = 0.02) • No difference in GMT for tetanus following booster for different treatments in JARD	NA NA NA NA	Lower transition B cell proportions in JARD (NA, *p* < 0.01) Lower transition B cell proportions in JARD on anti-TNF-alpha compared to JARD without anti-TNF-alpha and controls (NA, *p* < 0.01)
**Influenza**							
Aikawa et al. [18] Brazil 2013	38 JARD (25 JIA, 5 JDM, 3 JScle, 3 SLE, 2 vasculitis) (76%), median 7.0 y (range 2.6–9.0) 23 MTX, 12 steroids, 7 anti-TNF-alpha, 6 cyclosporine, 5 none, 1 azathioprine, 1 leflunomide^1^ 11 healthy (64%), median 7.8 y (range 3.2–8.9)	Single-centre, prospective cohort study (2B)	NA (*Butantan Institute/Sanofi Pasteur*) • A/California/7/2009(H1N1) pdm09 15 μg 2 (0; 21 d)	NA	• No difference in GMT, SCR and SPR between JARD and controls	JARD: pain 4 (11%) Controls: pain 2 (18%) JARD: headache 6 (16%), cough 4 (11%), fever 4 (11%), malaise 3 (8%), myalgia 2 (5%), rhinorrhoea 2 (5%), arthralgia 1 (3%) Controls: fever 1 (9%) None None	7 JARD (6 JIA, 1 SLE) did not seroconvert
Aikawa et al. [32] Brazil 2012	237 JARD (99 SLE, 93 JIA, 18 JDM, 11 JScle, 16 primary vasculitis) (66%), 14.8 y (SD 3.0) 90 steroids, 74 MTX, 43 azathioprine, 23 cyclosporine, 13 mycophenolate mofetil, 6 leflunomide, 3 cyclophosphamide^1^ 91 healthy (52%), 14.6 y (SD 3.7)	Single-centre, prospective cohort study (2B)	NYMCx-179A (*Butantan Institute/Sanofi Pasteur*) • A/California/7/2009(H1N1) pdm0915 μg	21 d	• Lower GMT in JARD (147.2 (95%CI 119.7–181.1) vs 250.8 IU/mL (95%CI 196.3–320.3), *p* = 0.01) • Lower SCR in JARD (74 (95%CI 69–80%) vs 96% (95%CI 91–100%), *p* < 0.01) • Lower SPR in JARD (81 (95%CI 77–86%) vs 96% (95%CI 91–100%), *p* < 0.01)	JARD: pain 43 (18%), itching 19 (8%), redness 9 (4%), swelling 3 (1%) Controls: pain 21 (23%), redness 21 (23%), swelling 2 (1%) JARD: headache 41 (17%), arthralgia 31 (13%), myalgia 27 (11%), rhinorrhoea 19 (8%), cough 16 (7%), fever 13 (6%), nasal congestion 13 (6%), sore throat 9 (4%), diarrhoea 8 (3%) Controls: headache 18 (20%), myalgia 6 (7%), cough 5 (6%), sorethroat 5 (6%), fever 3 (3%), nasal congestion 3 (3%), rhinorrhoea 3 (3%), arthralgia 2 (2%), diarrhoea 2 (2%) None None	Lower GMT in SLE compared to controls (91.1 (95%CI 66.0–125.8) vs 250.8 IU/mL (95%CI 196.3–320.3), *p* > 0.05) Lower GMT in JARD on azathioprine (NA, *p* = 0.02) and mycophenolate mofetil (NA, *p* = 0.01) compared to JARD on other treatments Lower SCR in SLE (64% (95%CI 54–73), *p* < 0.01), JIA (83% (95%CI 75–91), *p* = 0.01), JDM (78% (95%CI 59–97), *p* = 0.03) and primary vasculitis (75 (95%CI 54–96), *p* = 0.02) compared to controls (96% (95%CI 91–100%) Lower SCR in JARD on steroids compared to JARD without steroids (60 vs 83%, *p* < 0.01) Lower SCR in JARD on immunosuppressive drugs and steroids compared to JARD without immunosuppressive drugs and steroids (65 vs 78%, *p* = 0.04) Lower SPR in SLE compared to controls (73.7 (95%CI 65.0–82.4 vs 96% (95%CI 91–100%), *p* < 0.01)
Woerner et al. [33] Switzerland 2011	36 JARD (25 JIA, 3 uveitis, 2 inflammatory bowel disease, 2 chronic recurrent multifocal osteomyelitis, 1 JDM, 1 vasculitis, 1 SLE, 1 mixed connective tissue disease) (64%), median 13.8 y (range 2.3–16.0) 18 MTX, 10 anti-TNF-alpha, 8 anti-TNF-alpha + MTX 16 children with diabetes mellitus or cystic fibrosis (50%), median 11.9 y (7.8–15.1)	Single-centre, prospective cohort study (2B)	Inflexal V^®^(*Crucell Switzerland*) 2007/2008 • A/Solomon/3/2006(H1N1) • A/Wisconsin/67/2005(H3N2) • B/Malaysia/2506/2004 2008/2009 • A/Brisbane/59/2007(H1N1) • A/Brisbane/10/2007(H3N2) • B/Florida/4/2006 7.5 μg (> 3 y) or 15 μg (< 3 y + 1st dose) 1, 2 (< 3 y + 1st dose) (0; 1 m)	1–2 m	• No difference in GMT, SCR and SPR for any of the 3 strains between JARD and controls	JARD: pain or tenderness 5 (14%) Controls: pain or tenderness 2 (13%) JARD: fever, headache or myalgia 4 (12%) Controls: fever, headache or myalgia 1 (7%) None NA	No difference GMT, SCR and SPR for different treatments
Ogimi et al. [37] Japan 2011	49 JARD (23 JIA, 12 SLE, 6 JDM, 2 mixed connective tissue disease, 2 Kawasaki, 2 Takayasu arteritis, 1 Crohn’s disease, 1 Wegener granulomatosis) (71%), 12.1 y (SD 4.8) 14 steroids, 7 MTX + steroids, 7 mycophenolate mofetil + steroids, 6 cyclosporine + MTX + steroids, 2 azathioprine + steroids, 2 cyclosporine, 2 mizoribine + steroids, 1 azathioprine + cyclosporine + MTX, 1 azathioprine + cyclosporine + steroids, 1 azathioprine + mizoribine + steroids, 1 azathioprine + MTX + steroids, 1 cyclosporine + mizoribine + steroids, 1 cyclosporine + mycophenolate mofetil + steroids, 1 infliximab + MTX + steroids, 1 MTX, 1 MTX + tacrolimus + steroids 36 healthy (36%), 8.6 y (SD 14.3)	Single-centre, prospective cohort study (2B)	TIV (*Kaketsuken*, *BIKEN)* • A/H1N1 (NA) • A/H3N2 (NA) • B (NA) 0.1 (< 1 y), 0.2 (1–5 y), 0.3 (6–12 y), 0.5 mL (> 13 y) 2 (7–28 d)	14–28 d	• Higher GMT for B strain in JARD^7^ (60.3 vs 23.8, *p* < 0.01) • No difference in GMT for A/H1N1 and A/H3N2 strain between JARD and controls • No difference in SCR for any of the 3 strains between JARD and controls	JARD: pain and swelling 1 (2%) Controls: pain and/or swelling 3 (8%) None None JARD 2 (4%)	
Kanakoudi**–**Tsakalidou et al. [27] Greece 2001	70 JARD (49 JIA, 11 SLE, 3 JDM, 3 systemic vasculitis, 2 connective tissue disease, 1 Behçet’s disease, 1 idiopathic recurrent pericarditis) (73%), 11.6 y (SD 4.5) 16 MTX + steroids, 16 steroids, 11 cyclosporine + steroids, 8 cyclosporine + MTX + steroids, 6 azathioprine + steroids, 5 MTX, 4 cyclosporine, 4 cyclosporine + MTX	Single-centre, prospective case series (4)	Fluarix^®^SB (NA) • A/Beijing/262/95(H1N1) • A/Sydney/5/97(H3N2) • B/Beijing/184/93 NA 1, 2 (4–8 y and low pre-vaccination GMT) (0; 1 m)	1 m	• GMT for A/H1N1 (492.0), A/H3N2 (1449.0) and B strains (112.0) in JARD • SPR for A/H1N1 (97%), A/H3N2 (100%) and B strains (80%) in JARD • No difference in GMT and SPR for any of the 3 strains between JIA and SLE • No difference in GMT and SPR for any of the 3 strains for different treatments	JARD: 3 pain (4%), redness 1 (1%) JARD: fever and convulsion 1 (1%)^8^, sore throat and cough 1 (1%) None None	Higher increase in seroprotection for A/H1N1 compared to B strain after the 2nd vaccination in JARD with non-protective titre prior to 2nd vaccination (68% vs 5% (*p* < 0.01)) 15 JARD (13 JIA, 1 SLE, 1 systemic vasculitis) did not reach seroprotection
**Measles, rubella**							
Ingelman-Sundberg et al. [40] Sweden 2016	50 JARD (46 JIA, 1 erythema nodosum with arthritis, 1 JDM, 1 mixed connective tissue disease, 1 polyarteritis nodosa) (64%) median age JARD on MTX vs anti-TNF-alpha + MTX vs none 12.2 y (range 6.0–16.0) vs 13.1 y (range 2.9–18.3) vs 13.8 y (range 4.3–16.0) 15 etanercept, 10 MTX, 8 adalimumab + MTX, 8 none, 7 infliximab + MTX, 2 golimumab + MTX (some on additional steroids) 31 healthy (32%), median 11.5 y (range 2.4–17.7)	Single-centre, cross-sectional study (2C)	MMR (NA) NA ≥ 1 doses^6^ (1st dose before age 18 m)	NA	• No difference in GMT for measles and rubella between JARD with/without booster and healthy with/without booster	NA NA NA NA	Lower transition B cell proportions in JARD (NA, *p* < 0.01) Lower transition B cell proportions in JARD on anti-TNF-alpha compared to JARD without anti-TNF-alpha and controls (NA, *p* < 0.01) Low avidity range for rubella in 1 JARD (NA, *p* = 0.02)^9^
**Varicella**							
Speth et al. [24] Germany 2018	14 JARD (11 JIA, 2 JDM, 1 microscopic polyangiitis) on HIIS (71%), median 9.7 y (range 2.7–17.8) 2 etanercept, 2 MTX, 1 abatacept + leflunomide, 1 adalimumab + MTX, 1 anakinra + leflunomide + steroids, 1 anakinra + MTX + steroids, 1 etanercept + leflunomide + steroids, 1 etanercept + steroids, 1 leflunomide, 1 leflunomide + tocilizumab, 1 MTX + tocilizumab, 1 mycophenolate mofetil 9 JIA on LIIS (89%), median 8.3 y (range 1.8–17.8) 9 MTX	Single-centre, prospective cohort study (2B)	Varilrix^®^(*GlaxoSmithKline)* 10^3.3^ PFU 2 (42 d (LIIS), 3 m (HIIS))	1–3 m	• No difference in GMT between JARD on HIIS and JARD on LIIS	JARD on HIIS 1 (7%): NA JARD on LIIS 1 (11%): NA JARD on HIIS: arthralgia 1 (7%), elevated temperature 1 (7%), headache 1 (7%), vomiting 1 (7%) JARD on LIIS: arthralgia 3 (33%) None None	Low IgG levels (< 200 mIU/mL) in 2 JARD (1 on mycophenolate mofetil, 1 on leflunomide and abatacept) after 2nd vaccination
Groot et al. [25] Netherlands 2017	49 JARD (39 JIA, 5 JDM, 5 JScle) (53%) 1 vs 2 doses 5.0 y (range 2.0–15.0) vs 3.5 y (range 2.0–17.0) 25 MTX, 16 MTX + steroids, 2 cyclosporine + MTX, 2 leflunomide + MTX, 1 abatacept + MTX, 1 adalimumab + MTX, 1 azathioprine + MTX, 1 etanercept + MTX, 1 MTX + penicillamine 18 healthy (50%) median 8.5 y (range 3.0–18.0)	Single-centre, prospective cohort study (2B)	Oka strain (*Biken*) > 10^3^ PFU 1 if 2nd dose: Varilrix® (NA) 10^3.3^ PFU 1	28–42 d after 1st dose	• No difference in GMT between JIA, JDM or JScle and controls	None JIA: elevated temperature (< 38 °C) and vesicular rash 2 (3%) JScle: elevated temperature (< 38 °C) and vesicular rash 1 (20%) Controls: fever 1 (6%) None JIA: 3 (8%)	1 JARD (on abatacept) did not reach seroprotection after 2nd dose Higher GMT in JARD with 2 doses compared to JARD and controls with 1 dose (NA, *p* < 0.01) No difference in GMT for different treatments
				12 m after 1st dose	• No difference in GMT between JIA, JDM, JScle and controls		
Pileggi et al. [39] 2010 Brazil	25 JARD (17 JIA, 4 JDM, 2 JScle, 1 vasculitis) (52%), median 7.2 y (range 2.0–19.0) 12 MTX, 8 MTX + steroids, 3 cyclosporine + MTX + steroids, 1 leflunomide + MTX + steroids, 1 MTX + penicillamine + steroids 18 healthy (NA), median 9.3 y (range 3.0–18.0)	Single-centre, prospective cohort study (2B)	Oka strain (NA) 0.5 mL 1	28–42 d	• No difference in GMT and SPR between JARD and controls	None JARD: fever 1 (4%), rash 3 (12%) Controls: fever 1 (6%) None None	2 JARD who did not reach seroprotection developed varicella
				12 m	• SPR in JARD (80%)		

*95%CI *95% confidence interval*, ANA* antinuclear antibody, *anti-IL* anti-interleukin, *anti-TNF-alpha* anti-tumour necrosis factor alpha, *d* days, *DT* diphtheriae-tetanus vaccine, *DTP* diphtheria-tetanus-pertussis vaccine, *ERA* enthesitis-related arthritis, *GMT* geometric mean titre, *HAV* hepatitis A virus, *HBV* hepatitis B virus, *HIIS* high-intensity immunosuppression, *HPV* human papillomavirus, *IQR* interquartile range, *IU* international unit, *IVIG* intravenous immunoglobulins, *JARD* juvenile autoimmune rheumatic disease, *JDM* juvenile dermatomyositis, *JIA* juvenile idiopathic arthritis, *JScle* juvenile scleroderma, *LIIS* low-intensity immunosuppression, *m* month, *MMR* mumps-measles-rubella vaccine, *MTX* methotrexate, *NA* not available, *NSAID* non-steroidal anti-inflammatory drug, *PFU* plaque forming units, *RU* relative unit, *SCR* seroconversion rate, *SD* standard deviation, *SLE* systemic lupus erythematosus, *SPR* seroprotection rate, *SSZ* sulfasalazine, *TIV* trivalent influenza vaccine, *y* year

^1 ^Some children were receiving more than one drug

^2 ^Time after initiation of adalimumab (mean duration between last dose and sampling in children with ERA 7.2 (SD 3.2) vs healthy children 8.4 years (SD 2.0))

^3 ^Summary of all reactions occurring after 1st, 2nd, and 3rd vaccination in 128 JDM and 112 controls

^4 ^Numbers for children and controls not specified separately

^5 ^Higher use of immunosuppressive drugs in polyarticular onset JIA compared with oligoarticular JIA (80 vs 42% (*p* < 0.01))

^6 ^Children divided into a vaccinated group without booster (3 DTP, 1 MMR dose) and a group with booster vaccination (> 3 DTP, > 1 MMR)

^7^ Elevated GMT before vaccination in JARD compared to controls (19.7 vs 10.8 (*p* < 0.01))

^8 ^Child with epilepsy

^9 ^‘High avidity’ defined as > 60%, ‘borderline avidity’ as 40–59%, ‘low avidity’ as < 40%

**Table 3:** Risk of bias summary of studies included in the review

**Reference**	**Publication year**	**Study type**	**Confounding**	**Selection** **bias**	**Misclassification** **bias**	**Performance** **bias**	**Attrition** **bias**	**Detection** **bias**	**Reporting** **bias**
Maritsi et al. [43]	2019	CS	−	−	−	−	−	−	−
Grein et al. [42]	2020	CS	−	+	−	+	+	−	−
Guissa et al. [23]	2012	CS	−	−	−	−	−	−	−
Maritsi et al. [13]	2017	CS	−	−	−	−	−	−	−
Erguven et al. [35]	2011	CS	−	−	−	−	+	−	−
Kasapçopur et al. [16]	2004	CS	−	−	−	−	−	−	−
Heijstek et al. [44]	2014	CS	−	−	−	+	+	−	−
Brunner et al. [41]	2020	CSS	−	−	−	−	−	−	−
Heijstek et al. [30]	2012	CSS	+	+	−	−	−	−	−
Camacho − Lovillo et al. [12]	2017	CS	−	−	−	−	+	−	−
Aikawa et al. [22]	2013	CS	+	−	−	−	−	−	−
Carvalho et al. [46]	2013	CS	−	−	−	+	+	−	−
Dell’Era et al. [31]	2012	CS	−	−	−	+	−	−	−
Shinoki et al. [29]	2012	CS	+	−	−	−	−	−	−
Toplak et al. [28]	2012	CS	+	+	−	−	+	−	−
Stoof et al. [20]	2014	CS	+	−	−	−	−	−	−
Zonneveld-Huijssoon et al. [26]	2007	CS	−	−	−	−	−	+	−
Aikawa et al. [19]	2015	CS	−	−	−	−	−	−	−
Farmaki et al. [38]	2010	CS	+	−	−	−	−	−	−
Heijstek et al. [45]	2013	RCT	−	−	−	−	+	−	−
Borte et al. [14]	2009	CCS	−	+	−	+	−	−	−
Aytac et al. [34]	2011	CS	−	+	−	+	−	−	−
Miyamoto et al. [36]	2011	CCS	+	−	−	−	−	−	−
Kashef et al. [15]	2008	CCS	−	−	−	+	−	−	−
Campos et al. [21]	2013	CS	−	+	−	−	−	−	−
Barbosa et al. [47]	2012	RCT	−	−	−	−	−	−	−
Belderok et al. [17]	2013	CS	+	−	−	−	−	−	−
Heijstek et al. [48]	2013	CS	−	−	−	+	−	−	−
Ingelman-Sundberg et al. [40]	2016	CSS	+	−	−	+	−	−	−
Aikawa et al. [18]	2013	CS	−	−	−	−	+	−	−
Aikawa et al. [32]	2012	CS	+	+	−	−	−	−	−
Woerner et al. [33]	2011	CS	+	−	−	−	−	−	−
Ogimi et al. [37]	2011	CS	+	+	−	−	−	−	−
Kanakoudi-Tsakalidou et al. [27]	2001	Cs	−	−	−	−	−	−	−
Speth et al. [24]	2018	CS	+	−	−	−	+	−	−
Groot et al. [25]	2017	CS	+	−	−	−	−	−	−
Pileggi et al. [39]	2010	CS	−	−	−	−	−	−	−

*Cs* case series, *CS* cohort study, *CCS* case–control study, *CSS* cross-sectional study, *RCT* randomised controlled trial

## Results

Overall, 56 geometric mean antibody titres (GMTs) were measured in children with JARD on immunosuppressive treatment, of which 19 (34%) were lower, six (11%) higher, and 31 (55%) similar in these children compared to control children [12–16, 18–26, 28–34, 36–40, 43–45, 47, 48]. Of the 39 seroprotection rates (SPRs) measured, 10 (26%) were lower, two (5%) higher, and 27 (69%) similar in the two groups [12, 13, 16, 18, 19, 21–23, 29–35, 38, 39, 42, 43, 46]. Of the 27 seroconversion rates (SCRs) measured, nine (33%) were lower, two (8%) higher, and 16 (59%) similar in children with JARD compared with control children [12, 13, 18, 19, 21–23, 28, 29, 31–33, 37, 46].

### Enthesitis-related arthritis

#### Measles, rubella

One study investigated the persistence of specific antibodies after two doses of measles and rubella vaccination in 41 children with enthesitis-related arthritis (ERA) 1 and 3 years after the initiation of adalimumab and compared it with 149 healthy children [43]. At both timepoints, the GMTs were lower in children with ERA for measles and rubella compared with healthy children of similar age, while there was no difference in SPRs. No difference in GMTs or SPRs was found between children on adalimumab only and children who were on additional methotrexate (MTX) or sulfasalazine. The study provided no information about the safety of the vaccines.

### Juvenile dermatomyositis

Two studies investigated the immunogenicity and safety of vaccines in children with juvenile dermatomyositis (JDM) [23, 42]. The studies investigated human papilloma virus (HPV) and influenza vaccines in 72 children with JDM and 116 controls.

#### HPV

One study investigating the immunogenicity and safety of three doses of a HPV16/HPV18 vaccine in 42 children with JDM and 35 healthy children, 1 and 6 months after vaccination [42]. No difference in SPRs for both serotypes was detected 1 month after the last vaccination. Six months after the last vaccination, SPR for both serotypes was 94% in children with JDM. SPRs were not specified for healthy children. No difference in SPRs was found between children on different treatment regimens (steroids, hydroxychloroquine, MTX, azathioprine, cyclosporine, or mycophenolate mofetil) at either time point. No SAEs were observed. One child with JDM was reported to have a worsening in disease activity 6 months after vaccination.

#### Influenza

One study investigated the immunogenicity and safety of one dose of influenza vaccination (A/H1N1 strain) in 30 children with JDM and 81 healthy children, 21 days after vaccination [23]. A lower SCR was found in children with JDM. In contrast, no difference in GMT or SPR was detected. Separate results for different types of immunosuppressive treatment were not specified. No severe SAEs or worsening in diseases activity were reported.

### Juvenile idiopathic arthritis

A total of 18 studies investigated the immunogenicity and safety of 10 different vaccines (hepatitis A virus (HAV), hepatitis B virus (HBV), HPV, diphtheria, tetanus, influenza, meningococcus C (MenC), measles-mumps-rubella (MMR), pneumococcal polysaccharide, and conjugated vaccine) in 1555 children with JIA on immunosuppressive treatment, 4118 healthy children, and 14 children with non-rheumatic diseases [12–14, 16, 19, 20, 22, 26, 28–31, 35, 38, 41, 44–46].

### HAV, HBV

Two studies investigated the immunogenicity and safety of two doses of HAV vaccination in 130 children with JIA and 143 healthy children [13, 35]. One study found a lower GMT against HAV in children with JIA compared to healthy children 1 and 12 months after vaccination, but no difference in SCR or SPR, while the other study found a lower SPR in children with JIA 2 months after vaccination. The first study reported a worsening in disease activity in 15 (18%) children with JIA (in two after the first dose and in 13 after the second dose after a mean of 8 months) [13]. None of them had a worsening in disease activity during the first 3 months after vaccination. No SAEs were reported in the two studies.

The second study investigated the immunogenicity of three doses of HBV vaccine in 39 children with JIA and 41 healthy children and found a lower GMT and SPR in children with JIA [16]. No difference in GMTs was found between children on steroids and those on MTX. No SAEs were reported.

#### HPV

One study investigated the immunogenicity and safety of three doses of a HPV16/18 vaccine in 68 children with JIA and 55 healthy controls [44]. No difference in GMT was found. No difference in GMTs or antibody avidity was found between children on MTX and those on anti-tumour-necrosis-factor (TNF)-alpha blockers, anti-interleukin (IL)-1 blockers, leflunomide, and mycophenolate mofetil. One child on MTX did not reach seroprotection. No worsening in disease activitiy was reported. In 14 children with JIA and one healthy child SAEs were reported, many of them were elective hospitalisations or surgeries (for details see Table 2).

#### Diphtheria, tetanus

Two studies assessed the immunogenicity of diphtheria and tetanus vaccination in 429 children with JIA and 2176 healthy children [30, 41]. One study found lower GMTs and SPRs for diphtheria and tetanus in children with JIA compared to healthy children [30]. In the other study, no control children were included, the SPR for diphtheria in children with JIA was 90%, and for tetanus 100% [41]. No difference in GMTs or SPRs was found between children on abatacept, MTX, steroids, and anti-TNF-alpha blockers [30, 41]. Neither study provided information about SAEs or worsening in disease activity.

#### Influenza

Six studies investigated the immunogenicity and safety of one or two doses of influenza vaccines in 292 children with JIA, 154 healthy children, and 14 children with non-rheumatic diseases [12, 22, 28, 29, 31, 46]. Five studies used a trivalent influenza vaccine (TIV) and one study an influenza vaccine with an A/H1N1 strain only. The GMTs were lower against at least one strain in two studies [28, 31], the SCRs were lower against at least one strain in four studies [22, 28, 31, 46], and the SPRs were lower for at least one strain in two studies (see Tables 1 and 2) [31, 46]. Most studies did not report differences in specific antibody responses between different treatment regimens [12, 22, 29]. However, one study reported that children on anti-TNF-alpha blockers had lower SCR and SPR against A/H1N1 compared to children on leflunomide, MTX, steroids, and cyclosporine [46]. Only one study specified SAEs; one child with JIA on etanercept needed a hospitalisation for fever and coxalgia 1 day after vaccination [31]. Eighteen children with JIA reported a worsening in disease activity 7 days to 6 months after vaccination [12, 28, 31].

#### MenC

Two studies investigated the immunogenicity of one dose of MenC vaccination in 361 children with JIA and 1527 healthy children [20, 26]. One study found no difference in GMT between children with JIA and healthy ones [20]. The other study, which compared different immunosuppressive treatments in children with JIA, found a lower GMT against MenC in children treated with MTX, sulfasalazine, etanercept, infliximab, or cyclosporine compared to children on non-steroidal anti-inflammatory drugs or without treatment [26]. No information concerning SAEs was provided. No worsening in disease activity was reported.

#### Pneumococcus

One study each investigated the immunogenicity and safety of one dose of 23-valent pneumococcal polysaccharide vaccine (PPV23) and two doses of 7-valent pneumococcal conjugate vaccine (PCV7) in 27 and 63 children with JIA, respectively [19, 38]. Both studies compared the antibody response to pneumococcus in children with JIA on MTX and cyclosporine with children with JIA on the former treatment plus additional adalimumab or etanercept. The study using the PPV23 vaccine found no difference in GMTs, SCRs, or SPRs for serotypes 4, 6B, 9 V, 14, 18C, 19F, and 23F between the two groups [19]. The study using the PCV7 vaccine found lower GMTs but not SPRs for serotypes 4, 14, and 23F in on adalimumab or etanercept compared to those on steroids or cyclosporine [38]. However, no difference in GMTs was found between children on adalimumab and those on etanercept [38]. One child on etanercept developed pneumococcal pneumonia (serotype not specified) requiring hospitalisation 5 months after vaccination [19]. In one child with JIA on additional adalimumab or etanercept, a worsening in disease activity was reported [38].

#### MMR

Three studies, including one RCT, investigated the immunogenicity of MMR vaccination in 546 children with JIA and 2198 healthy children [14, 30, 45]. In the RCT, both groups included children with JIA on immunosuppressive treatment who had been previously been vaccinated with MMR, but only one group was randomised to receive a MMR booster [45]. In the other two studies, either two doses of MMR or a monovalent measles vaccine followed by MMR were given [14, 30].

One study reported a higher GMT against measles in children with JIA compared to healthy children [30]. However, the children with JIA were older than the healthy children; therefore, likely more of them had received an MMR booster, which was given at nine years of age. Another study reported a lower GMT against measles in children with JIA after receiving MTX for 6 months initiated more than 4 years after the second dose of MMR compared to healthy children [14]. But again the children were from different age groups. The children with JIA had a mean age of 16 years compared to the healthy children with a mean age of 11 years. Therefore, there was a larger time interval between vaccination and measuring vaccine antibody responses in children with JIA. Only one study measured SPRs for measles and reported a higher SPR in children with JIA compared to healthy children in the group vaccinated with two doses of MMR but not in the group vaccinated with a monovalent measles vaccine followed by a dose of MMR [30]. However, as mentioned above some of the healthy children, who were younger, likely did not yet receive two doses of the MMR vaccine. The RCT reported an increase in GMT in the children with JIA who received an MMR booster 12 months after the vaccine [45].

Two studies compared GMTs against mumps in children with JIA compared to healthy children [14, 30]. One found a lower GMT against mumps in children with JIA, while the other did not find a difference in GMTs. The one study which measured SPRs for mumps reported no difference in children with JIA compared to the healthy controls when receiving two doses of MMR but a lower SPR for mumps in the children who received a monovalent measles vaccine followed by one dose of MMR [30]. The RCT reported an increase in GMT against mumps in the children with JIA who received an MMR booster 12 months after vaccination [45].

One study did not detect a difference in rubella specific GMTs between children with JIA and healthy children, while another study found a lower GMT in children with JIA [14, 30]. The study which measured SPRs against rubella, reported a lower SPR in children with JIA compared to healthy children after one dose of monovalent measles vaccine followed by MMR but not in the children who received two doses of MMR [30]. The RCT reported a higher GMT in children with JIA receiving a MMR booster compared to children with JIA without the booster 12 months after vaccination [45].

No difference in GMTs against measles, mumps, or rubella was found between children on MTX and children on steroids, anti-TNF-alpha blockers, anti-IL-1 blockers, or leflunomide [30, 45].

Only two of the three studies evaluated the safety of the MMR vaccine; no worsening in disease activity was reported in either of the studies [14, 45]. Only the RCT reported SAEs; five children who received a booster and eleven children in the control group were reported to have SAEs [45]. Most of them were elective hospitalisation and surgeries, unlikely related to the vaccination. No disease due to infections with vaccine viruses was observed [45].

### Systemic lupus erythematosus

Five studies including 236 children with systemic lupus erythematosus (SLE) and 228 healthy children investigated the immunogenicity and safety of five different vaccines (HBV, tetanus, influenza, measles, and varicella (VZV)) [15, 21, 34, 36, 47].

#### HBV

One study investigated the immunogenicity and safety after three doses of an HBV vaccine in 20 children with SLE and 24 healthy children [34]. A lower GMT was found in children with SLE, while the SPR was similar in both groups. Separate results for different types of immunosuppressive treatment were not reported. No SAEs were reported. Three children with SLE were reported to have a worsening in disease activity, one child 1 month after the first dose and the other two children 1 month after the second dose.

#### Tetanus

Two studies investigated the specific antibody response after five doses of tetanus vaccine in 70 children with SLE and 74 healthy children [15, 36]. In one study, a lower GMT was found in children with inactive SLE (but not active SLE) compared to healthy children [36]. The other study did not find a difference in GMTs between children with SLE and healthy ones [15]. Separate results for different types of immunosuppressive treatment were not reported. During the study, four SAEs occurred in children with SLE; all were disseminated infections requiring hospital admission unlikely related to the vaccine (disseminated varicella infection, primary peritonitis, meningitis and pneumonia) [15]. No information concerning worsening in disease activity was provided in either study [15, 36].

#### Influenza

One study investigates the immunogenicity of one dose of an A/H1N1 influenza vaccine in 118 children with SLE and 102 healthy children [21]. A lower GMT, SCR, and SPR were found in children with SLE. Children on a higher steroid dose did more frequently not seroconvert compared to those on a lower dose (18.0 vs 10.5 mg per day). No SAEs or worsening in disease activity were reported.

#### Measles

One retrospective study investigated the specific antibody response after measles vaccination in 30 children with SLE with different immunosuppressive treatments (chloroquine, steroids, azathioprine, cyclosporine, cyclophosphamide, and MTX) and 28 healthy children [36]. No difference in GMTs was found between children with active SLE, inactive SLE and healthy children. Separate results for different types of immunosuppressive treatment were not reported. No information concerning SAEs and worsening in disease activity was provided.

#### VZV

One RCT investigated the immunogenicity and safety of one dose of VZV vaccine in 28 children with SLE on either chloroquine, steroids, azathioprine, or MTX and 28 healthy children [47]. No difference in GMT for children with JIA compared to healthy ones was found 1, 6, and 12 months after vaccination. Separate results for different types of immunosuppressive treatment were not reported. Furthermore, no SAEs or worsening in disease activity were reported.

###  Other JARDs

Eleven studies including 667 children with different JARDs, 254 healthy children, and 16 children with non-rheumatic diseases investigated the immunogenicity and safety of HAV, HBV, HPV, tetanus, influenza, measles, rubella, and VZV vaccines [17, 18, 24, 25, 27, 32, 33, 37, 39, 40, 48].

#### HAV, HBV

One study investigated the immunogenicity of two doses of a combined HAV and HBV vaccines in 78 children with JARD, including 71 (91%) children with JIA [17]. A positive SCR was reached for HAV and HBV in 100% and 93%, respectively. No difference in GMTs for different treatments was found. No information concerning SAEs and worsening in disease activity was provided.

#### HPV

One study investigated the immunogenicity of three doses of a HPV in 12 children with JARD (6 JDM, 6 SLE) and 49 healthy children [48]. No difference in GMTs between JARD and controls were found 1 and 6 months after vaccination. One child with JDM without immunosuppressive treatment did not seroconvert. Separate results different types of immunosuppressive treatment were not reported. No information concerning SAEs was provided. One child with JDM was reported to have a worsening in disease activity 1 month after the second vaccination.

#### Tetanus

One study investigated the immunogenicity of more than three doses of a tetanus vaccine in 50 children with JARD (including 46 (92%) children with JIA) and 31 healthy children [40]. A lower GMT was detected in children with JARD on MTX with or without anti-TNF-alpha blockers compared to children with JARD without treatment or healthy children. No information concerning SAEs and worsening in disease activity was provided.

#### Influenza

Five studies investigated the immunogenicity of influenza vaccination in 430 children with JARD, 138 healthy children, and 16 children with non-rheumatic diseases [18, 27, 32, 33, 37]. Three studies used a TIV and two studies a single A/H1N1 strain vaccine. Two studies did not find differences in GMT, SCR, or SPR between children with JARD and controls [18, 33]. One study found a lower GMT, SCR, and SPR for the A/H1N1 strain in children with JARD compared to healthy children [32]. In contrast in another study, a higher GMT for the B strain in children with JARD compared to healthy children was found, while there was no difference in SCR for all three strains [37]. One study reported a lower GMT in children on azathioprine, mycophenolate mofetil, and steroids compared to children on cyclosporine, leflunomide, or cyclophosphamide [32]. No SAEs were reported [18, 27, 32, 33, 37]. In one study a worsening in disease activity was reported in two children with JARD (one child with JIA, one with Takayasu arteritis) 2 weeks after vaccination [37].

#### Measles, rubella

One study investigated the specific antibody responses against measles and rubella after a minimum of one dose of MMR in 50 children with JARD (46 children with JIA) on MTX or MTX plus anti-TNF-alpha blockers and 31 healthy children [40]. No difference in GMTs for measles or rubella was found between children with JARD and healthy children. Children with JARD on anti-TNF-alpha blockers had a lower proportion of transition B cells compared with those without anti-TNF-alpha blockers and controls. No information concerning SAEs and worsening in disease activity was provided.

#### VZV

Three studies investigated the immunogenicity of one or two doses of VZV vaccination in 97 children with JARD on different immunosuppressive treatments and 36 healthy children [24, 25, 39]. No difference in GMT or SPR was found in any of the three studies. No SAEs were reported. Separate results for different types of immunosuppressive treatment were not reported. One study reported a worsening in disease activity in three children with JIA 4 to 6 weeks after vaccination [25].

## Discussion

Our systematic review shows that vaccines in children and adolescents with JARDs on immunosuppressive treatment are safe and immunogenic. Overall, a decreased specific antibody response was reported in one-third (26–33%) of all measurements (GMT, SPR, or SCR). However, it is important to take into consideration the timing of the measurements. For example, a study, which measured antibodies to HAV/HBV vaccine in children with JARD, reported that the initial response after one dose was low, but after receiving a second dose, almost all children with JARD seroconverted [17]. This stresses the importance for the completion of vaccination schedules, especially in high-risk children, such as children with JARDs.

Furthermore, due to the lower vaccine response found in approximately one-third of children with JARDs on immunosuppressive treatment, additional booster doses can be offered to optimise vaccine efficacy in these children. Two studies showed an accelerated antibody loss in children with JIA [6, 26]. In the included RCT, which randomised children with JIA to either receive or not receive a booster dose of MMR, a higher proportion of children who did not receive a booster were not seroprotected [45]. This demonstrates the importance of booster doses in children with JARDs. A pro-active approach to detect insufficient antibodies levels in children with JARDs might be a valuable tool to optimise the timing booster doses. Similarly, pre-travelling antibody measurements could be useful, for example, for HAV in children who are travelling to high incidence countries.

A further main concern in children with inflammatory or autoimmune diseases, including JARD, is that vaccines may trigger an onset or worsening in disease activity. For example, it has previously been reported that HBV vaccination might trigger the onset of an underlying inflammatory or autoimmune rheumatic disease [49] or that HPV vaccination could trigger the onset of SLE [50]. Many studies exclude children with active JARD to avoid potential worsening in disease activity [34]. Only one study included in the review, compared the immunogenicity of a measles and tetanus vaccine in children with active and inactive SLE [36]. The study reported a higher immunogenicity of the vaccines in children with active compared to inactive SLE. However, the children in the latter group were older than the ones with active SLE, which likely explains some of this difference.

In our review, ten studies reported children with worsening in disease activity after vaccination [12, 13, 25, 28, 31, 34, 37, 38, 42, 48]. However, often it could not be differentiated between the vaccines as a trigger for the worsening of disease or other possibilities causes, such as changes or non-compliance to the immunosuppressive treatment [31]. Furthermore, many of the JARDs are characterised by an intermittent and relapsing course even without triggers. As many care providers prefer to vaccinate these children in a stable phase and often defer vaccination until such a phase is reached, the chance of a relapse due to the normal course of the disease might be higher after vaccination, which can then be misinterpreted as a relapse triggered by the vaccine. Although, worsening in disease activity might be a more of a concern after live attenuated vaccines, only one study reported a worsening in disease activity in three of 39 children with JIA, 4 to 6 weeks after VZV vaccination [25]. In contrast, one of the studies reported a decrease in number of affected joints in children with JARD after VZV vaccination [39]. Another study included in the review, investigated the association of autoantibodies and disease activity after influenza vaccination in children with JIA, and did not find an association [28]. Importantly, it should be noted and communicated that vaccine-preventable diseases, for example mumps, measles, and rubella, can also trigger an activation or exacerbation of the underlying inflammatory or autoimmune JARD [51, 52]. Overall, there is increasing evidence suggesting that vaccines do not induce significant worsening of underlying disease [8, 53].

Not only for a worsening in disease activity, but also for SAEs, it is difficult to proof a correlation. Many of the SAEs reported in the studies included in this review seem unlikely to be correlated to vaccination (e.g. elective hospitalisations and surgeries). As an example, two children with SLE have been reported to have deterioration in renal function within 18 months after HPV vaccinations [54]. However, it is not clear if this is an adverse event due to the vaccination or rather illustrate the natural progression of the disease itself. As both cases were diagnosed with state four nephritis before vaccination, an evolution of the disease itself seems more likely. In another study, a febrile convulsion was reported as a severe adverse event after influenza vaccination in a child with JARD. However, this child was known for epilepsy [27]. In one study, a vesicular rash was reported in three of 49 children with JARD on immunosuppressive treatment with MTX and anti-TNF-alpha blockers [25]. Another study reported that rashes after VZV vaccination were not more common in children with SLE on immunosuppressive treatment compared to healthy children [47]. None of the studies reported an infection due to attenuated vaccine viruses, and all recovered promptly without treatment. These results are, however, limited by most studies investigating live attenuated vaccine not reporting systemic reactions, severe adverse events, or worsening in disease activity [14, 30, 36, 40, 43].

Unfortunately, there is no study which investigated the immunogenicity and safety of yellow fever vaccination in children with JARDs on immunosuppressive treatment. A recent review from the European League Against Rheumatism concluded that yellow fever vaccination in adults with autoinflammatory rheumatic diseases should be avoided due to the risk of vaccine-induced yellow fever [55]. As yellow fever is circulating in many parts of the world, the safety and immunogenicity of the vaccine in children with JARDs is an urgent future research topic.

Furthermore, the current severe acute respiratory syndrome coronavirus type 2 (SARS-CoV-2) pandemic also highlights the urgent need to assess the immunogenicity and safety of new vaccines, such as messenger ribonucleic acid-based (mRNA) vaccines, in this patient group. Three recent studies showed that mRNA and viral vector-based SARS-CoV-2 vaccines are immunogenic and safe (no increased side effects or induction of disease flares) in adults with rheumatic diseases on immunosuppressive treatment [56–58]. However, specific antibody responses were lower in adults on immunosuppressive treatment compared to healthy controls, especially those on steroids, rituximab, mycophenolate mofetil, and abatacept [56, 58]. No data is currently available on the immunogenicity and safety of SARS-CoV-2 vaccine in children with rheumatic diseases on immunosuppressive treatment.

The strengths of our review are the systematic approach and the comprehensive literature search. The limitations are the heterogenicity of the included studies which precluded a meta-analysis, especially as vaccine responses were not always measured one month after vaccination (gold-standard) and sometimes the time interval between vaccination and measurement of responses even differed between cases and controls. Furthermore, the dose of the immunosuppressive treatment and the disease activity or severity was not specified in most of the studies. Many of the studies were underpowered, and not designed to show non-inferiority between children with JARD and controls; therefore, finding no difference between the groups does not imply equivalence. Moreover, we only evaluated antibody responses, as there are almost no studies which report on vaccine efficacy, and cellular or cytokine responses to vaccines.

In conclusion, vaccination in children with JARD on immunosuppressive treatment should be promoted and the importance for the completion of vaccination schedules should be stressed. Strategies to compensate for the lower vaccine responses or faster decline of antibodies include measuring antibody levels to determine the optimal timing for the administration of additional booster doses. Further studies including children with active JARD are needed for evidence-based guidelines to vaccination in these children.

## Supplementary Information

Below is the link to the electronic supplementary material.

Supplementary file1 data Search terms used for OVID search (titles also used as search terms) (DOCX 19 KB)

## Data Availability

N/A

## Abbreviations

ERA: Enthesitis-related arthritis
GMT: Geometric mean titre
HAV: Hepatitis A virus
HBV: Hepatitis B virus
HPV: Human papilloma virus
JARD: Juvenile autoimmune rheumatic disease
JDM: Juvenile dermatomyositis
JIA: Juvenile idiopathic arthritis
MenC: Meningococcus C
MMR: Measles-mumps-rubella
MTX: Methotrexate
PCV7: 7-valent pneumococcal conjugated vaccine
PPV23: 23-valent pneumococcal polysaccharide vaccine
RCT: Randomised controlled trial
SAE: Severe adverse event
SCR: Seroconversion rate
SLE: Systemic lupus erythematosus
SPR: Seroprotection rate
TIV: Trivalent influenza vaccine
VZV: Varicella

## Code availability

N/A
