# Supplementary material for: Immunogenicity and safety of routine vaccines in children and adolescents with rheumatic diseases on immunosuppressive treatment — a systematic review
Source: Eur J Pediatr. 2021 Dec 22;181(4):1329–62. doi: 10.1007/s00431-021-04283-w (PMC8692821; doi:10.1007/s00431-021-04283-w)
Supplement: Supplementary file 1 — Supplementary file1 data Search terms used for OVID search (titles also used as search terms) (DOCX 19 KB) [file 431_2021_4283_MOESM1_ESM.docx]

**Supplementary data** Search terms used for OVID search (titles also used as search terms)

| Immunosuppressive drugs | Immunosuppressive drugs | Children | Vaccination |
| --- | --- | --- | --- |
| abatacept | fluticason | adolescen* | attenuated vaccin* |
| actinomycin* | gemcitabin* | babies | bacillus calmette guerin vaccin* |
| adalimumab | glucocorticoids | baby | bacille calmette guerin vaccin* |
| alemtuzumab | glucocorticosteroid* | boy | bacterial vaccin* |
| alemtuzumab | golimumab | boys | BCG vaccin* |
| anakinra | guselkumab | child | chickenpox vaccin* |
| anti CTLA 4 | humira | childhood | cholera vaccin* |
| anti-cytotoxic t-lymphocyte antigen 4 | hydrocortisone | children | combined vaccin* |
| anti TNF alpha | hydroxychloroquin* | girl | conjugate vaccin* |
| Anti-tumour necrosis factor alpha | hydroxyurea | girls | diphtheria vaccin* |
| anti-CD-20 | idarubicin | infan* | diphtheria toxoid* |
| anti-CD20 | ifosfamide | juvenil* | diphtheria-tetanus vaccin* |
| anti-IL-1 | immunosupp* | kinder | diphtheria-tetanus-acellular pertussis vaccin* |
| anti-IL-2 | immunosuppressive agents | kinder-aged | diphtheria-tetanus-pertussis vaccin* |
| anti-IL-6 | immunosuppressive treatment* | kindergarten* | H1N1 vaccin* |
| anti-IL1 | infliximab | kinders | haemophilus influenza b vaccin* |
| anti-IL2 | interleukin 1 receptor antagonist protein | neo-nat* | haemophilus vaccin* |
| anti-IL6 | interleukin-1beta | neonat* | HAV vaccin* |
| anti-interleukin 1 | ipilimumab | new-born* | HBV vaccin* |
| anti-interleukin 2 | irinotecan | newborn* | hepatitis A vaccin* |
| antirheumatic agents | ixekizumab | paediatric* | hepatitis B vaccin* |
| apremilast* | janus kinase inhibitors | pediatric* | herpes zoster vaccin* |
| asparaginase | leflunomid | pre-schooler* | HPV vaccin* |
| atlizumab | lenalidomide | preschooler* | immunisation |
| azathioprine | melphalan | school-age* | immunization |
| baricitinib | mepolizumab | school-boy* | inactivated vaccin* |
| basiliximab | mercaptopurine | school child* | influenza vaccin* |
| belatacept | methotrexate | School girl* | japanese encephalitis vaccin* |
| belimumab | methylprednisolone | schoolage* | measles vaccin* |
| betamethasone | mitomycin | schoolboy* | measles-mumps-rubella vaccin* |
| bevacizumab | mitoxantrone | schoolchild* | meningococcal vaccin* |
| biological products | mmf | schoolgirl* | mumps vaccin* |
| bleomycin | mtx | teen | mycobacterium bovis vaccin* |
| budesonide | muromonab-CD3 | teenage* | papillomavirus vaccin* |
| busulfan | mycophenolat mofetil | teens | pertussis vaccin* |
| canakinumab | mycophenolic Acid | toddler* | pneumococcal vaccin* |
| capecitabine | natalizumab | youth | polio vaccin* |
| carboplatin | ocrelizumab | youths | poliomyelitis vaccin* |
| certolizumab | omalizumab |  | poliovirus vaccin* |
| cetuximab | oxaliplatin |  | rabies vaccin* |
| chlorambucil | paclitaxel |  | rotavirus vaccin* |
| chloroquine | pemetrexed |  | rubella vaccin* |
| ciclosporin* | pimecrolimus |  | salmonella vaccin* |
| cisplatin | pirfenidon |  | tbe vaccin* |
| cladribine | pomalidomid* |  | tetanus toxoid* |
| corticosteroid* | predniso* |  | tetanus vaccin* |
| cortisol | procarbazin* |  | tick borne encephalitis vaccin* |
| cyclophosphamide | rituximab |  | typhoid fever vaccin* |
| cyclosporine | ruxolitinib |  | typhoid vaccin* |
| cytarabine | sarilumab |  | typhoid-paratyphoid vaccin* |
| cytostatic agents | secukinumab |  | vaccin* |
| dacarbazine | siltuximab |  | vaccines |
| daclizumab | SSZ |  | viral hepatitis vaccin* |
| dactinomycin | sulphasalazine |  | viral vaccin* |
| daunorubicin | tacrolimus |  | whooping cough vaccin* |
| dexamethasone | temozolomid |  | yellow fever vaccin* |
| dimethyl fumarate | teriflunomid |  |  |
| disease modifying anti rheumatic drugs | thalidomide |  |  |
| DMARD | tildrakizumab |  |  |
| docetaxel | tocilizumab |  |  |
| doxorubicin | tofacitinib |  |  |
| dupilumab | topoisomerase Inhibitors |  |  |
| epirubicin | topotecan |  |  |
| etanercept | trastuzumab |  |  |
| etoposide | ustekinumab |  |  |
| fingolimod | vedolizumab |  |  |
| fingolimod hydrochloride | Vidarabine |  |  |
| fludarabine | vinblastine |  |  |
| fluorouracil | vincristine |  |  |
